# A Unique ATPase, ArtR (PA4595), Represses the Type III Secretion System in *Pseudomonas aeruginosa*

**DOI:** 10.3389/fmicb.2019.00560

**Published:** 2019-03-21

**Authors:** Weina Kong, Mengmeng Dong, Rong Yan, Qingqing Liang, Huiqun Zhang, Wei Luo, Yani Zhang, Haihua Liang, Kangmin Duan

**Affiliations:** ^1^Key Laboratory of Resources Biology and Biotechnology in Western China, Ministry of Education, Faculty of Life Sciences, Northwest University, Xi’an, China; ^2^Department of Oral Biology and Department of Medical Microbiology, Rady Faculty of Health Sciences, University of Manitoba, Winnipeg, MB, Canada

**Keywords:** *Pseudomonas aeruginosa*, type III secretion system, PA4595, ArtR, REG sub-family, gene regulation

## Abstract

*Pseudomonas aeruginosa* is an important human pathogen which uses the type III secretion system (T3SS) as a primary virulence factor to establish infections in humans. The results presented in this report revealed that the ATP-binding protein PA4595 (named ArtR, a Regulator that is an ATP-activated Repressor of T3SS) represses T3SS expression in *P. aeruginosa*. The expression of T3SS genes, including *exoS, exoY*, *exoT*, *exsCEBA*, and *exsD-pscB*-*L*, increased significantly when *artR* was knockout. The effect of ArtR on ExsA is at the transcriptional level, not at the translational level. The regulatory role and cytoplasm localization of ArtR suggest it belongs to the REG sub-family of ATP-binding cassette (ABC) family. Purified GST-tagged ArtR showed ATPase activity *in vitro*. The conserved aspartate residues in the dual Walker B motifs prove to be essential for the regulatory function of ArtR. The regulation of T3SS by ArtR is unique, which does not involve the known GacS/A-RsmY/Z-RsmA-ExsA pathway or Vfr. This is the first REG subfamily of ATP-binding cassette that is reported to regulate T3SS genes in bacteria. The results specify a novel player in the regulatory networks of T3SS in *P. aeruginosa*.

## Introduction

*Pseudomonas aeruginosa* is a major Gram-negative opportunistic pathogen capable of causing a variety of infections in immuno-compromised individuals and cystic fibrosis patients (Richards et al., 2000; Kerr and Snelling, 2009). It uses an arsenal of virulence factors to establish wide range of human infections. Among these virulence factors, the type III secretion system (T3SS) plays a critical role in *P. aeruginosa* pathogenicity (Coburn et al., 2007; Engel and Balachandran, 2009). The T3SS system is composed of the type III secretion and translocation machinery, regulators, effectors and effector-specific chaperones. T3SS allows direct delivery of several bacterial effector proteins into eukaryotic host cells (Ghosh, 2004). Four effectors ExoS, ExoT, ExoU, and ExoY have been identified in different strains of *P. aeruginosa* (Frank, 1997; Yahr et al., 1998). The translocated effectors are important to the bacterium’s successful evasion of phagocytosis and dissemination (Kurahashi et al., 1999; Roy-Burman et al., 2001; Vance et al., 2005).

T3SS gene expression in *P. aeruginosa* is induced by the contact of host cells and low-calcium conditions (Frank, 1997; Vallis et al., 1999). All T3SS genes are under the direct transcriptional control of ExsA, a member of the AraC-type DNA binding protein (Hovey and Frank, 1995). ExsA auto-regulates its own expression by activating transcription of the *exsCEBA* operon. In addition, the T3SS is coordinately regulated through other regulatory pathways such as cAMP/Vfr and Gac/Rsm systems. Virulence factor regulator (Vfr) is a cAMP-dependent regulator of transcription. The Vfr regulon consists of hundreds of genes including quorum sensing, type IV pili, type II secretion system, and the T3SS (Wolfgang et al., 2003). Recent study shows that Vfr can regulate the expression of T3SS via directly activating *exsA* transcription (Marsden et al., 2016). The Gac/Rsm regulatory pathway is another central pathway in controlling T3SS expression. The global posttranscriptional regulatory protein RsmA can activate T3SS and repress biofilm formation, hence controlling the switch between acute and chronic infections (Brencic and Lory, 2009; Irie et al., 2010). Two small regulatory RNAs, RsmY and RsmZ, bind RsmA to antagonize its function. The GacS/GacA two-component regulatory system exclusively controls the expression of RsmY and RsmZ (Brencic et al., 2009). These studies indicate that the T3SS of *P. aeruginosa* is fine-tuned by complicated environmental cues and internal regulatory networks.

ATP binding cassette (ABC) transporters constitute one of the largest of paralogous protein families with a diversity of physiological functions and they are highly conserved in all species from microbes to human (Davidson et al., 2008). Although most known ABC ATPases such as the Classes 1 and 3 are involved in transmembrane transport of molecules, the superfamily also includes soluble ATPases (Class 2) performing diverse non-transport functions (Bouige et al., 2002; Davidson et al., 2008). The REG subfamily of Class 2 ABC ATPases has several members identified in bacteria (Bouige et al., 2002; Davidson et al., 2008). Proteins in this subfamily are devoid of transmembrane domains and consist of two ABC ATPase domains fused in tandem (Kerr, 2004). ChvD, in *Agrobacterium tumefaciens* has been found to be involved in the regulation of virulence gene expression (Liu et al., 2001). So far, the most investigated bacterial member of the REG subfamily is Uup in *Escherichia coli*. It is involved in transposon precise excision and bacterial competitiveness and ATP hydrolysis is essential for the function of the Uup (Hopkins et al., 1983; Reddy and Gowrishankar, 1997; Murat et al., 2006, 2008). Secondary structure and NMR resonance assignments of the C-terminal DNA-binding domain (CTD) of Uup protein suggests that Uup CTD is a DNA-binding coiled coil motif (Carlier et al., 2012a,b). There are three other REG subfamily proteins (YheS, YbiT, and YjjK) in *E. coli* (Murat et al., 2008). Recent studies demonstrate that the EttA (YjjK) protein in *E. coli* gates ribosome entry into the translation elongation cycle through a nucleotide-dependent interaction sensitive to ATP/ADP ratio (Boel et al., 2014; Chen et al., 2014).

In our search for regulatory genes of the T3SS in *P. aeruginosa*, a probable ABC ATPase, named ArtR, was found to repress the transcription and secretion of T3SS effectors in PAO1. ArtR is predicted to be a soluble ABC ATPase and has 74.77% similarity to the REG subfamily protein EttA in *E. coli* (Boel et al., 2014; Chen et al., 2014). So far, no member in this subfamily has been reported to be involved in regulation of T3SS. In this study, our results suggest that ArtR may not be involved in transport process, and that the ArtR protein could inhibit the transcription but not the translation of T3SS proteins, which seems to be functionally different from EttA in *E. coli*. These results add a novel regulatory factor to the T3SS expression in *P. aeruginosa* and provided a new thread for further study of the REG subfamily ATPases.

## Results

### Identification of a Putative ABC ATPase That Influences T3SS Transcription and Effector Secretion

**FIGURE 1:**
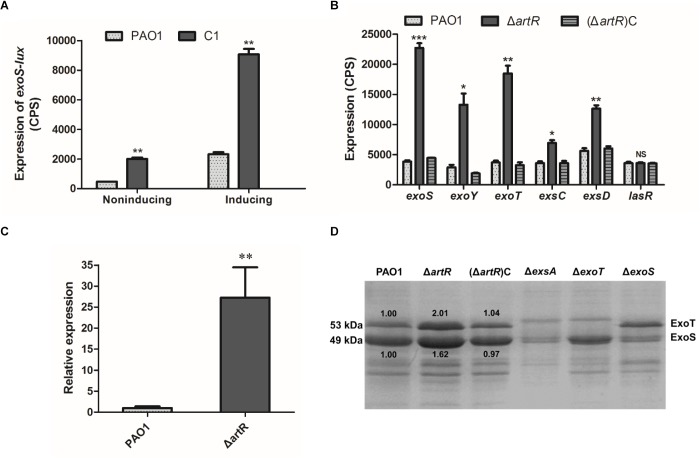
Effect of *artR* mutation on T3SS expression and secretion. **(A)** A CTX-*exoS* reporter fusion integrated on the chromosome was used to measure expression levels of *exoS* under T3SS non-inducing or inducing conditions in C1 mutant strain and wild-type PAO1 after 6 h growth. Error bars indicate one standard deviation. ^∗∗^*p* < 0.01 compared to the wild-type based on Student’s *t*-test. **(B)** Strains carried reporter plasmids pKD-*exoS*, pKD-*exoY*, pKD-*exoT*, pKD-*exsC*, or pKD-*exsD* were cultured under T3SS inducing conditions. Promoter activities were measured after 6 h growth. Error bars indicate one standard deviation. NS, *p* > 0.05, ^∗^*p* < 0.05, ^∗∗^*p* < 0.01, or ^∗∗∗^*p* < 0.001 compared to the wild-type. **(C)** The RT-qPCR analysis of the normalized mRNA expression level of the *exoS* gene in the Δ*artR* strain under T3SS inducing conditions. ^∗∗^*p* < 0.01. **(D)** Culture supernatants of various strains after 6 h of growth in T3SS inducing medium were concentrated and analyzed by SDS-PAGE, followed by staining with Coomassie blue. The bands of approximately 53 and 49 kDa were corresponding to ExoT and ExoS respectively. The relative grayscale value of ExoS and ExoT bands were quantified using ImageJ.

T3SS is a primary virulence factor in *P. aeruginosa*, and alterations in T3SS expression often concur with disease progression. Previously, we have screened the *P. aeruginosa* genome for genes that affect T3SS expression as part of an attempt to investigate the regulatory mechanisms that govern *P. aeruginosa* infection. A transposon insertion library of wild-type PAO1 containing an *exoS*-*lux* transcriptional reporter on its chromosome was constructed and mutants with altered promoter activity of T3SS effector gene *exoS* were collected. A hybrid sensor kinase PA1611 was found to control biofilm formation and T3SS through direct interaction with RetS (Kong et al., 2013; Bhagirath et al., 2017). Among the rest of the transposon mutants that regulate T3SS, one mutant C1 with disrupted PA4595, which we designated as ArtR, a Regulator that is an ATP-activated Repressor of T3SS, was found to have significantly elevated *exoS* expression under both T3SS non-inducing and inducing conditions (Figure 1A). The *artR* gene encodes a putative soluble ABC ATPase. No ABC ATPase has previously been implicated in T3SS expression. Further characterization of this mutant was carried out to investigate the genetic mechanisms underlying the connection between ArtR and T3SS.

To verify the observed effect of *artR* disruption, a gene knockout mutant of *artR* was first constructed. A Gm^r^-*lacZ* cassette was inserted within *artR*, and the resultant mutant was named as Δ*artR*. The knockout strain Δ*artR* was also complemented by inserting an intact copy of *artR* with its promoter region at the *attB* site on the chromosome. The resultant strain was designated as (Δ*artR*)C. After introduction of reporter plasmid pKD-*exoS* into Δ*artR*, (Δ*artR*)C and the wild-type PAO1 respectively, the expression of *exoS* was compared. As shown in Figure 1B, the knockout mutant showed significant elevated *exoS* expression, and the level of *exoS* expression was restored to wild-type levels in the complementation strain, confirming that ArtR was responsible for the changed *exoS* expression.

To test if ArtR further influences the expression of other T3SS genes, the expression of *exoY*, *exoT*, *exsCEBA* and *exsD-pscB*-*L* was also compared in Δ*artR*, (Δ*artR*)C and PAO1. As shown in Figure 1B, the expression of these T3SS genes increased significantly in the *artR* knockout mutant and the levels of expression were restored to wild-type levels in the complementation strain. As a negative control, no difference in the expression level of quorum sensing regulatory gene *lasR* was observed between Δ*artR* mutant and PAO1. The result indicates that ArtR had a significant effect on T3SS.

To verify the transcriptional differences of these effector genes caused by *artR* deletion, RT-qPCR analysis of the mRNA level of the *exoS* gene in the Δ*artR* strain was carried out and the results indicate that the normalized *exoS* mRNA levels were greatly increased in the mutant comparing with that in the wild-type (Figure 1C). This confirms that *artR* had an effect on T3SS at the transcriptional level.

T3SS transcription is coupled with the effector secretion process in *P. aeruginosa* (Yahr and Wolfgang, 2006). To assess whether the increased T3SS transcription was reflected on the secretion of T3SS effectors, we grew these strains in T3SS inducing media and surveyed culture supernatants for secreted T3SS effectors. The Δ*exsA* mutant which does not produce any effectors was used as a control. SDS-PAGE analysis of supernatants of PAO1 showed bands of approximately 53 and 49 kDa, corresponding to ExoT and ExoS respectively, while the mutant strain Δ*exoT* and Δ*exoS* did not have the respective band corresponding to ExoT and ExoS (Kong et al., 2013). In contrast, Δ*artR* secreted more effectors than the wild-type strain (Figure 1D). We also quantified the change in ExoS and ExoT secretion in Figure 1D using ImageJ. The secretion of ExoS and ExoT was indeed increased in Δ*artR*. These results demonstrate that *artR* mutant indeed had increased T3SS secretion. Further verification of the effect of *artR* on T3SS was carried out by examining ExoS production in the cells of Δ*artR*, (Δ*artR*)C and PAO1 using Western blot. The results confirmed an increase of ExoS in the mutant (Supplementary Figure S1).

### The Function of ArtR Is Neither Associated With the Adjacent Transmembrane Transport nor Involved in the Regulation via Vfr or RsmA

Most bacterial ABC ATPases in the transport systems are encoded within operons with membrane proteins, whereas *artR* is a single gene without association with any membrane protein. The operon (PA4594-PA4591) adjacent to *artR* in PAO1 genome is also predicted to encode components of ABC transporter.

**FIGURE 2:**
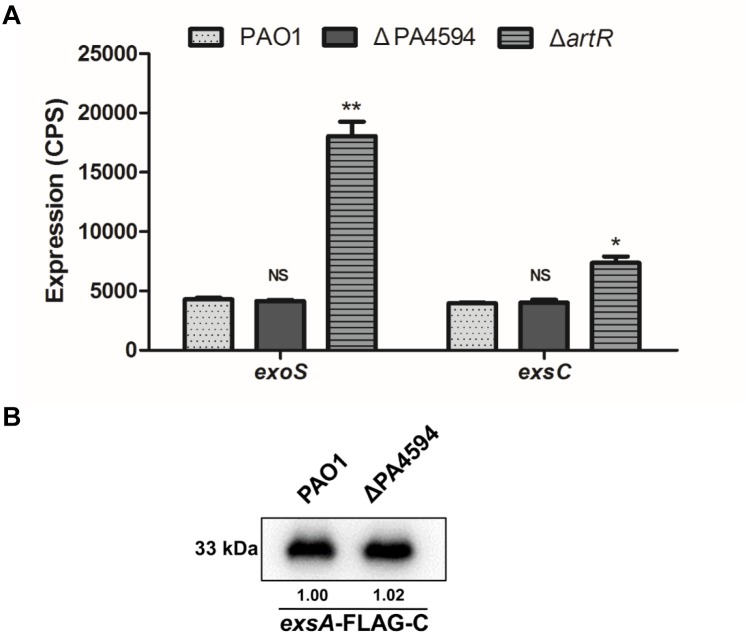
Expression of T3SS genes in ΔPA4594. **(A)** Comparison of the expression of *exoS* and *exsC* in PAO1, Δ*artR* and ΔPA4594. Strains carried reporter plasmids pKD-*exoS* or pKD-*exsC* were cultured under T3SS inducing conditions. Promoter activities were measured after 6 h growth. Unlike *artR*, deletion of the adjacent ABC transporter gene PA4594 had no effect on *exoS* expression. Error bars represent one standard deviation from the mean. NS*p* > 0.05, ^∗^*p* < 0.05, or ^∗∗^*p* < 0.01 compared to the wild-type. **(B)** The levels of ExsA-FLAG in PAO1 and ΔPA4594. Indicated strains carrying an *exsA*-FLAG-C fusion were grown to an OD_600_ of 1.0 in LB with 5 mM EGTA and 20 mM MgCl_2_. The whole-cell extracts from the designated strains were subjected to SDS-PAGE separation and subsequent immuno-blotting. The bands of approximately 33 kDa were corresponding to ExsA-FLAG. The relative grayscale value of the ExsA-FLAG bands was quantified using ImageJ and shown underneath.

To examine whether ArtR is associated with this operon and whether it functions as one of the components of the transporter to affect T3SS, a gene knockout mutant of PA4594 was constructed and the resultant strain was named ΔPA4594. Comparison of *exoS* and *exsCEBA* expression levels in ΔPA4594, Δ*artR*, and PAO1 indicates that PA4594 had no influence on the expression of *exoS* and *exsCEBA* (Figure 2A), indicating that the function of ArtR is distinct from the adjacent ABC transporter. Further comparison of the ExsA levels in the mutant and the wild type was performed by Western blot analysis of the ExsA-FLAG-C in these strains. As shown in Figure 2B, no difference was observed in the FLAG-tagged ExsA between them, confirming the adjacent transporter was not involved.

**FIGURE 3:**
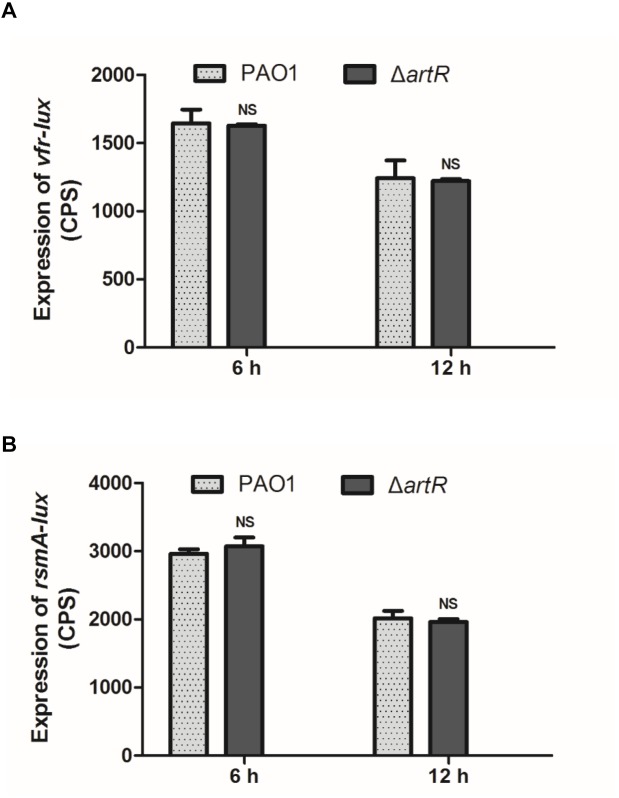
Expression of *vfr* and *rsmA* in PAO1 and Δ*artR*. **(A)** The expression of *vfr* in PAO1 and Δ*artR*. **(B)** The expression of *rsmA* in PAO1 and Δ*artR*. Strains carried reporter plasmids pKD-*vfr* or pKD-*rsmA* were cultured in LB medium. Promoter activities were measured after 6 and 12 h growth. Error bars represent one standard deviation from the mean. NS*p* > 0.05 compared to the wild-type.

Vfr is a cAMP-dependent DNA-binding protein that directly activates *exsA* transcription which is the central regulator of T3SS gene expression (Marsden et al., 2016). To investigate the regulation pathway of T3SS by ArtR, we measured *vfr* promoter activity in Δ*artR* mutant. No difference in the levels of *vfr* expression between Δ*artR* mutant and the wild-type strain was observed (Figure 3A).

The small protein RsmA in the Gac/Rsm regulatory pathway is a pleiotropic global posttranscriptional regulator in *P. aeruginosa.* The function of RsmA is regulated by two antagonists small RNA RsmY/RsmZ (Brencic and Lory, 2009; Brencic et al., 2009). RsmA regulates the T3SS via modulation of ExsA (Intile et al., 2014). We constructed an *rsmA* promoter-*lux* fusion (*rsmA*-*lux*) and measured its activity in the wild-type and Δ*artR* strains. The result, as shown in Figure 3B, indicates no difference in *rsmA* expression between these two strains. Consistently, neither the *rsmY* promoter-*lux* reporter nor *rsmZ* promoter-*lux* reporter showed changed activity in Δ*artR* comparing with the wild-type (data not shown).

### The Regulatory Effect of ArtR on ExsA Is at the Transcriptional Level, Not at the Translational Level

ArtR is a putative soluble ABC ATPase which belongs to the REG subfamily. There are three other REG subfamily proteins in *P. aeruginosa*. To deduce ArtR action, a phylogenetic analysis of the REG subfamily ATPases of *P. aeruginosa* and *E. coli* K-12 was performed. ArtR showed stronger sequence similarity (74.77%) to EttA than to other soluble ABC proteins (Supplementary Figures S2, S3). Both EttA and ArtR harbor two tandem ABC ATPase domains but lack the C-terminal DNA-binding domain (CTD) of Uup (Supplementary Figure S4). The EttA protein in *E. coli* has been demonstrated to regulate protein synthesis probably in response to cellular energy status (Boel et al., 2014; Chen et al., 2014).

Given that the transcription of T3SS was affected by ArtR, we tested whether ArtR regulates T3SS via ExsA, the master regulator of T3SS. We reasoned that unless the effect is directly on individual T3SS genes such as *exoS, exoT, exoY*, and *exsC*, which is unlikely, any regulatory effect on T3SS would be reflected in the amount of ExsA protein in the cells. To differentiate the effect of ArtR on the transcription of *exsCEBA* or on the translation of ExsA, we cloned two *exsA*-FLAG fusions with different promoter regions. In the *exsA*-FLAG-C fusion, the promoter region of the *exsCEBA* operon and the *exsA* coding region were fused with FLAG tag. In the *exsA*-FLAG-A fusion, the *tac* promoter and the *exsA* coding region were fused with FLAG tag (Li et al., 2013). We transferred these two *exsA*-FLAG fusions into the *artR* mutant and PAO1. The expression levels of the ExsA-FLAG were then tested under T3SS inducing conditions.

**FIGURE 4:**
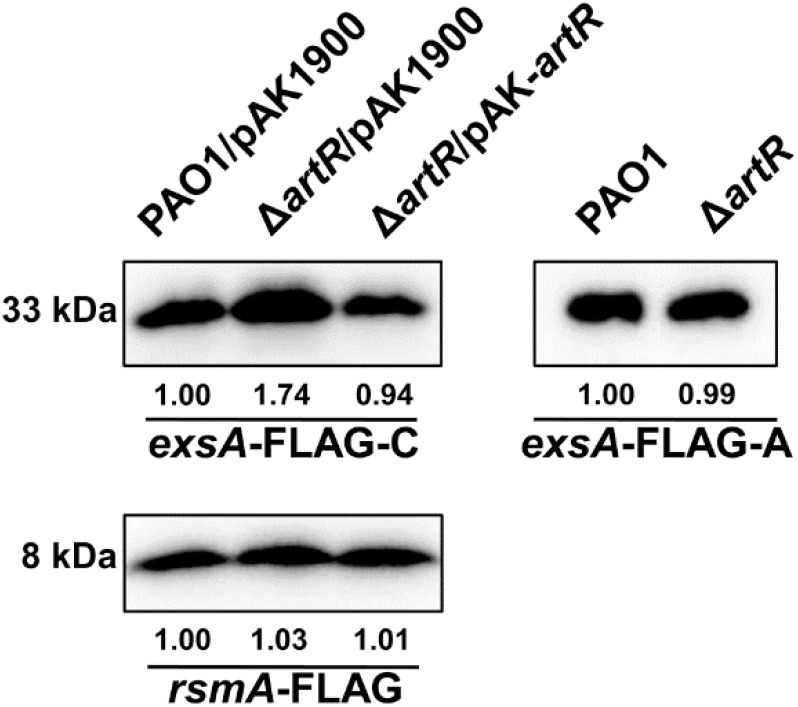
ArtR doesn’t influence the translation process of ExsA. Western-blotting showed the expression levels of ExsA-FLAG and RsmA-FLAG in PAO1 and the *artR* mutant. The *exsA*-FLAG-C fusion contained the promoter region of the *exsCEBA* operon and the *exsA* coding region. The *exsA*-FLAG-A fusion carried the *tac* promoter and the *exsA* coding region. The *rsmA*-FLAG fusion contained the promoter region and coding region of *rsmA*. The pAK1900 plasmid carrying the entire *artR* gene and the promoter region of *artR* was used for the complementation of Δ*artR*. The bands of approximately 33 and 8 kDa were corresponding to ExsA-FLAG and RsmA-FLAG respectively. The relative grayscale value of ExsA-FLAG and RsmA-FLAG bands were quantified using ImageJ.

As shown by results from the *exsA*-FLAG-C construct, the ExsA-FLAG protein level in the *artR* mutant was higher than that in PAO1 as expected. However, when the T3SS promoter was replaced by the *tac* promoter, the ExsA-FLAG protein levels were similar in both PAO1 and the *artR* mutant (Figure 4), demonstrating that the translation process of ExsA was not affected by the *artR* mutation.

We also constructed an *rsmA*-FLAG fusion with its own promoter region. As shown in Figure 4, RsmA-FLAG protein levels in PAO1 and the *artR* mutant were similar, suggesting that ArtR doesn’t affect either the transcription or translation of RsmA. These results indicate that despite the homology between ArtR and EttA, they seem to function through different mechanisms. The regulation of T3SS by ArtR does not appear to be through controlling the translation of T3SS protein.

To acquire a global view of the regulatory effect of ArtR in *P. aeruginosa*, we have performed RNA-seq analysis to compare the gene expression profiles between PAO1 and the *artR* mutant. The genes that were differentially expressed are listed in Supplementary Table S3. One of the most notable changes in the *artR* mutant was the significant higher expression level of the T3SS genes in the mutant. While total of 11 genes showed increased levels of expression in the mutant, 8 of them are T3SS genes, including *exoS, exoT, exoY, pcrV, exsC* and *exsA, pscC and pscF*. The RNA-seq result confirms the specific effect of ArtR on the transcription of T3SS genes. The effect of ArtR is apparently not a general effect on protein translation as how EttA functions in *E. coli*.

### The Highly Conserved Aspartate Residues in Both Walker B Motifs Are Essential for the Function of ArtR

**FIGURE 5:**
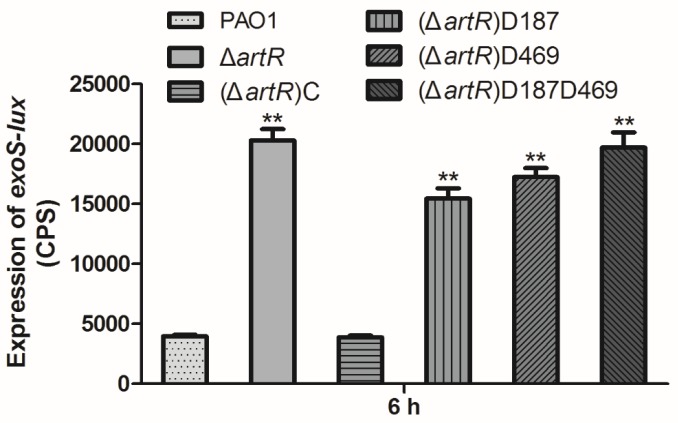
Expression of *exoS* in PAO1 and *artR* point mutants. Strains carried reporter plasmid pKD-*exoS* were cultured under T3SS inducing conditions. Promoter activities were measured after 6 h growth. Error bars represent one standard deviation from the mean. ^∗∗^*p* < 0.01 compared to the wild-type based on Student’s *t*-test.

REG subfamily ATPases are consists of two ABC ATPase domains fused together. Each Walker B motif in the ATPase domain harbors a highly conserved aspartate residue which is important for ATP hydrolysis catalytic cycle of ABC ATPases (Kuhnau et al., 1991; Shyamala et al., 1991). To determine whether the conserved Asp residues are required for the function of ArtR, we introduced D187E and D469E single amino acid residue substitutions as well as double residue substitutions into ArtR. These derivatives of ArtR were then introduced into the *artR* knockout mutant, and the expression of *exoS* in these point mutants was compared. As shown in Figure 5, the constructs carrying either D187E or D469E or both abolished the complementation ability of ArtR to restore the *exoS* expression level. Only the complete ArtR was able to complement. Clearly, both Asp residues play critical roles in the function of ArtR.

### Subcellular Localization of ArtR

**FIGURE 6:**
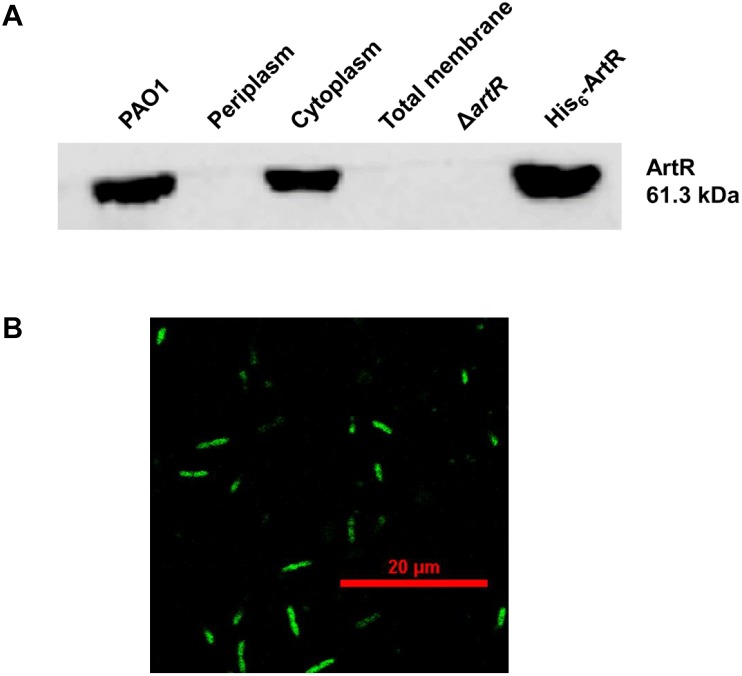
Subcellular localization of ArtR. **(A)** Localization of ArtR in the cell fractionations analyzed by Western blot. Immunoblot of crude extracts probed with the polyclonal antibody (1:3,000) directed against ArtR protein. Cells of a 50 ml culture in LB were fractionated. Specimens of the whole cell preparation of PAO1, periplasm, cytoplasm, total membrane, the total cell extract of Δ*artR* (negative control), and His_6_-ArtR (positive control) were separated by SDS-PAGE and probed by immunoblotting. **(B)** The localization of ArtR-GFP in PAO1. *P. aeruginosa* PAO1 carrying pME6032-*artR-gfp* was cultured with 0.5 mM IPTG at 37°C for 4 h. The cells were examined and imaged by Nikon A1 confocal laser scanning microscopy.

ATP-binding cassette ATPase are often embedded in the cell membrane. However, many REG subfamily ATPases are found in the cytoplasm in recent years (Murat et al., 2006, 2008; Boel et al., 2014; Chen et al., 2014). ArtR protein is also predicted by PSORT as a cytoplasm protein. To verify the cellular location of ArtR, solubilized His_6_-ArtR protein was purified to prepare polyclonal antibodies used to localize the ArtR protein in PAO1 cell fractions by Western blot. As shown in Figure 6A, ArtR was detected in the whole cell preparation and the cytoplasmic fraction of PAO1. No band was observed in the periplasmic or the membrane fractions of PAO1 or in the total cell extract of Δ*artR*, confirming that ArtR protein is a cytosolic protein.

To verify the cytoplasmic location of ArtR, we also expressed GFP-tagged ArtR and examined the cellular distribution of ArtR-GFP in PAO1 by confocal laser scanning microscopy. As shown in Figure 6B, the GFP tagged ArtR is uniformly distributed in the cell, supporting its cytoplasmic location.

### ArtR Has *in vitro* ATPase Activity

**FIGURE 7:**
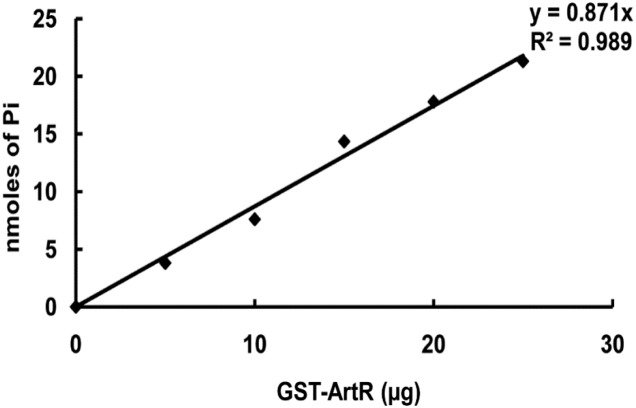
ATPase activity of purified GST-ArtR protein. The ATPase activity of GST-ArtR protein was detected by assaying the amount of inorganic phosphate liberated from ATP using NaH_2_PO_4_ as a standard. Purified GST-ArtR protein (0–25 μg) was added into the reaction mixtures. The reaction was started by adding 2 mM ATP and terminated after 1 h by the addition of the Malachite Green reagent. Purified GST tag was used as negative control and no ATPase activity was detected.

To verify the ATPase activity of ArtR, we carried out *in vitro* analysis. GST-tagged ArtR protein was purified by using a high-affinity GST resin column (Supplementary Figure S5). Spontaneous ATPase activity of ArtR was tested by analyzing the amount of inorganic phosphate liberated from the ATP substrate. The release of Pi occurred in a protein concentration-dependent manner (Figure 7) when purified GST-ArtR protein was incubated with ATP, demonstrating that purified GST-ArtR had ATPase activity and capable of hydrolyzing ATP.

## Discussion

*Pseudomonas aeruginosa* is a human pathogen that causes serious and often life-threatening infections. T3SS is a virulence factor in *P. aeruginosa* that is extremely important for its successful establishment of infection and evasion of phagocytosis. It is essential to understand the pathways and controlling mechanisms of the T3SS. T3SS also provides a novel target for the development of new therapeutic strategies against *P. aeruginosa* infections. Although dozens of genes implicated in the regulation of T3SS in *P. aeruginosa* have been identified in the past 10 years, important regulators of the T3SS are still to be discovered.

In this study, a probable ABC ATPase ArtR was found to repress T3SS expression at the level of transcription. The *artR* gene encodes a soluble ABC ATPase which belongs to the REG subfamily (Supplementary Figures S2, S3). There are only four similar soluble ATPases in *P. aeruginosa* which are respectively homologous to Uup, YheS, YbiT, and EttA in *E. coli*. So far, none of them has been investigated nor found to be connected with T3SS. Unlike most other ABC ATPases involving in transport process, *artR* is a single gene without membrane protein encoding genes in its operon. Our data demonstrate that ArtR has function different from the adjacent PA4594 operon which is predicted to encode components of ABC transporter. The influence of ArtR on the expression of T3SS is not due to the potential transport function. Neither the effect of ArtR on the T3SS was through the known pathways, GacS/A-RsmY/Z-RsmA-ExsA or Vfr pathways.

The regions of ArtR corresponding to Walker motif B were aligned with the Uup protein in *E. coli*. The invariant aspartate residues that are important for the catalytic cycle of ABC ATPases in Uup correspond to D187 and D469 in ArtR. The results presented here indicate that both residues D187 and D469 play critical roles in the function of ArtR. The negative regulation of ArtR on T3SS is dependent on its ATPase activity. Recombinant GST-ArtR protein acquired was found to display ATPase activity.

Members of REG subfamily display significant functional diversity in eukaryotes and prokaryotes. Although a few of them have been reported to participate in gene expression regulation, their exact mechanism of action and the role of ATP hydrolysis in their function have remained unknown. The yeast protein GCN20 and its human ortholog ABC50 have been implicated in the control of mRNA translation (de Aldana et al., 1995; Tyzack et al., 2000). The information collected on prokaryote proteins is quite descriptive and does not afford a clue for their actual physiological role and mechanism of action. In *E. coli*, one of the REG subfamily protein, Uup, was found to be involved in transposon precise excision and bacterial competitiveness (Murat et al., 2006, 2008). This protein is demonstrated to be a cytoplasmic ABC protein. Analysis of Walker motif B mutants suggests that ATP hydrolysis at the two ABC domains is strictly coordinated and is essential for the function of Uup *in vivo* (Murat et al., 2006). In addition, Uup carries a C-terminal DNA-binding coiled coil motif (Carlier et al., 2012a,b). Another REG subfamily protein EttA in *E. coli* has been demonstrated to regulate protein synthesis potentially in response to cellular energy status (Boel et al., 2014; Chen et al., 2014). In this study, we performed a phylogenetic analysis of the REG subfamily ATPases in *P. aeruginosa* and *E. coli*. The sequence of ArtR was similar to EttA and both lack the C-terminal DNA-binding domain of Uup (Supplementary Figure S4). We constructed two *exsA*-FLAG fusions to test whether ArtR regulates T3SS via controlling the translational process of ExsA. The results demonstrated that the translation process of ExsA is not affected by the *artR* mutation (Figure 4). ArtR and EttA seem to have different functional mechanisms. EttA has been shown to inhibit protein translation process in *E. coli* (Boel et al., 2014; Chen et al., 2014). It gates ribosome entry into the translation elongation cycle through a nucleotide-dependent interaction sensitive to ATP/ADP ratio (Boel et al., 2014). Despite its similarity to EttA, ArtR seems to specifically regulate the transcription of T3SS genes. This is in contrast to the effect of EttA on protein translation, as indicated by the unaffected translational levels of ExsA in Δ*artR*. However, it has been suggested that EttA may act preferentially on the translation of specific target mRNAs (Boel et al., 2014). It is possible that the translation of an unknown component in the T3SS regulatory network is specifically affected by ArtR, which resulted in the changed transcription of T3SS genes. Considering the activity of T3SS is energy demanding, it makes sense to couple the T3SS with the energy status of the cell. Further study is needed to confirm such possibilities.

ArtR is found to be highly conserved in many bacteria through homology analysis, suggesting that the protein performs important functions in bacteria. This report reveals that ArtR is a unique component in the regulatory network of T3SS in *P. aeruginosa* and provides new path to further study the REG subfamily ATPases in bacteria.

## Experimental Procedures

### Strains, Plasmids and Growth Conditions

The bacterial strains and plasmids used in this study are listed in Supplementary Table S1. *P. aeruginosa* and *E. coli* were routinely grown on Luria-Bertani (LB) agar or in LB broth at 37°C unless otherwise specified. LB was used as T3SS non-inducing conditions and LB supplemented with 5 mM EGTA and 20 mM MgCl_2_ as T3SS inducing conditions. Antibiotics were used at the following final concentrations. For *P. aeruginosa*: gentamicin (Gm) at 50 μg/ml in LB or 150 μg/ml in *Pseudomonas* isolation agar (PIA), tetracycline (Tc) at 70 μg/ml in LB or 300 μg/ml in PIA, carbenicillin (Cb) at 250 μg/ml in LB and trimethoprim (Tmp) at 300 μg/ml in LB. For *E. coli*: kanamycin (Kn) at 50 μg/ml, ampicillin (Ap) at 100 μg/ml, Tc at 15 μg/ml, and Gm at 15 μg/ml in LB.

### Construction of Gene Expression Detecting System

The plasmid pMS402 carrying a promoterless *luxCDABE* reporter gene cluster was used to construct promoter*-luxCDABE* reporter fusions as reported previously (Duan et al., 2003). Promoter regions (including the intergenic region and partial coding region) of genes were PCR amplified respectively using high-fidelity *Pfu* DNA polymerase (Fermentas) and primers designed according to the PAO1 genome data (Stover et al., 2000). The promoter regions were cloned into the *Bam*HI-*Xho*I site upstream of the *lux* genes on pMS402. The resultant plasmids were transformed into PAO1 by electroporation. DNA manipulation, PCR, and transformation were performed following standard procedures. Cloned promoter sequences were confirmed by DNA sequencing.

Besides the plasmid-based reporter system, an integration plasmid CTX6.1 originated from plasmid mini-CTX-*lux* (Becher and Schweizer, 2000) was used to construct chromosomal fusion reporter system. This plasmid has all the elements required for integration, origin of replication, and tetracycline resistance. A DNA fragment of the pMS402-based reporters that contains the kanamycin resistance determinant, the multiple cloning sites, and the promoter-*luxCDABE* reporter was then ligated into CTX6.1. The plasmid generated was transferred into *E. coli* SM10-λ*pir* (Simon et al., 1983) and the integration strain was constructed through biparental mating as reported previously (Hoang et al., 2000; Liang et al., 2008).

Using these *lux*-based reporters, gene expression in liquid cultures was examined as counts per second (cps) of light production in a Synergy 2 Multimode Microplate Reader (BioTek). Measurements were taken every 30 min for 24 h. Bacterial growth was monitored at the same time by measuring the OD_600_ using the Microplate Reader.

### Screening for Regulatory Genes of the T3SS

The transposon mutagenesis library was constructed as previously described except some modifications (Kulasekara et al., 2005). Briefly, the donor strain *E. coli* SM10-λ*pir* containing pBT20 and the *P. aeruginosa* PAO1 carrying the reporter *exoS-lux* on its chromosome were grown on solid media overnight and the cells were collected and spotted on LB agar plate at a ratio of 2:1. After incubation for 2 h, the mixed culture was diluted and spread on PIA containing gentamicin. A transposon mutant library was constructed by picking 10,000 colonies grown on the selective plates. After overnight incubation, colonies with changed activities of *exoS-lux* under a LAS-3000 imaging system (FUJIFILM) were collected. Verification of the mutant phenotype was carried out in liquid cultures. The expression of *exoS* in collected mutants was examined in Synergy 2 Multimode Microplate Reader. The sites of transposon insertion in the selected mutants were determined by arbitrary primed PCR and subsequent sequencing of the PCR products (Friedman and Kolter, 2004; Liang et al., 2008).

### Construction of Gene Knockout Mutant

For gene knockout, the previously described *sacB*-based strategy was employed (Hoang et al., 1998). The regions of target genes were PCR amplified using primers listed in Supplementary Table S2. The PCR products were digested with restriction enzymes and cloned into pEX18Tc. The fragment containing Gm^r^-*lacZ* from the plasmid pZ1918-*lacZ*Gm (Schweizer, 1993) was then inserted into the target genes on pEX18Tc. *P. aeruginosa* knockout mutants were obtained following triparental mating between *E. coli* strain containing the helper plasmid pRK2013 (Ditta et al., 1980), the *P. aeruginosa* recipient and the *E. coli* donor harboring the pEX18Tc based knockout construct. The resultant mutants were verified by PCR the region containing the target gene and PCR products sequencing.

### Complementation of the *artR* Knockout Mutant

For the complementation experiments, the entire gene together with upstream promoter region of *artR* was integrated into the *attB* site on the mutant’s chromosome using the mini-CTX-*lacZ* system (Becher and Schweizer, 2000). The DNA fragment was PCR amplified using primers pEX-*artR*-S and pEX-*artR*-A (Supplementary Table S2). The product was cloned into mini-CTX-*lacZ*. The generated plasmid was transferred into *E. coli* SM10-λ*pir* and the complementary strain was obtained after biparental mating. The resultant strain was designated as (Δ*artR*)C.

### Construction of ArtR Point Mutations

The construction of the D187E mutation in the first Walker B motif of ArtR was accomplished by PCR using oligonucleotide primers containing the point mutation and an *Xho*I restriction site. The *Xho*I restriction site was generated from the base alterations at D187 without affecting adjacent codons. The DNA fragment upstream of codon D187 was amplified using primers pEX-*artR*-S containing a *Kpn*I site and *artR*D187-A containing an *Xho*I site; the downstream region of D187 was generated using the primers *artR*D187-S containing an *Xho*I site and pEX-*artR*-A containing a *Hin*dIII site (Supplementary Table S2). These two PCR fragments were cloned in turn into mini-CTX-*lacZ*. In the final construct, the aspartate residue D187 of ArtR was replaced by a glutamate residue. The same strategy was used to construct the D469E mutation in the second Walker B motif of ArtR. The primers used are listed in Supplementary Table S2. The D187E D469E double substitution mutations on ArtR were generated by swapping the wild-type fragment upstream of the D187E mutation with the fragment containing D469E mutation using the *Sph*I and *Hin*dIII restriction sites. All these mutations were verified by sequencing. The plasmids generated were used to complement of the *artR* knockout mutant as above and the resultant strains were designated as (Δ*artR*)D187, (Δ*artR*)D469, and (Δ*artR*)D187D469 respectively.

### RNA Extraction and Real-Time Quantitative PCR (RT-qPCR)

Total RNA was extracted from 500 μl fresh broth culture (OD_600_ = 1.0) by using the Trizol reagent. Genomic DNA was eliminated by RNase-free DNase I treatment after the isolation procedure. Then 1 μg RNA sample was reverse transcribed to cDNA using the qScript cDNA SuperMix Kit (Quanta). Real-time qPCR was performed in the LightCycler (MBI Lab Equipment) using PerfeCTa SYBR Green FastMix Kit (Quanta). The fold change of relative expression was showed by the value of 2^−ΔΔCt^. The primers specific for *exoS* and the housekeeping gene *proC* (Savli et al., 2003) are shown in Supplementary Table S2.

### T3SS Effector Secretion Assay

*Pseudomonas aeruginosa* strains were grown at 37°C in T3SS inducing conditions for 6 h. Following removal of the bacterial cells by centrifugation at 14,000 *g*, proteins in the supernatant were precipitated following addition of an equal volume of 100% trichloroacetic acid, washed with acetone, and pelleted. The proteins were re-suspended in sample buffer, separated by SDS-PAGE, and visualized with Coomassie stain (Goodman et al., 2004).

### *In vitro* Expression and Purification of ArtR Protein

ArtR protein was expressed as a glutathione S-transferase (GST) tagged fusion protein using the pGEX protein expression system (GE Healthcare). The DNA fragment covering the entire *artR* gene was PCR amplified using primers pGEX-*artR*-S and pGEX-*artR*-A (Supplementary Table S2). The PCR product was then cloned into pGEX-4T-1 to generate pGEX-ArtR which was verified by sequencing. pGEX-ArtR was transferred into *E. coli* strain BL21(DE3)pLysS for ArtR protein production. BL21(DE3)pLysS carrying pGEX-ArtR was grown at 37°C in LB broth supplemented with 100 μg/ml Ap. When the culture reached an OD_600_ of 0.5–1.0, isopropyl β-D-thiogalactopyranoside (IPTG) was added at a final concentration of 0.5 mM to induce protein expression. The cells were harvested 3 h later by centrifugation. The pellets from 50 ml of culture were re-suspended in 6 ml PBS supplemented with 1 mM phenylmethanesulfonyl fluoride (PMSF) and 1 mg/ml lysozyme. Cells were ruptured by sonication using a Hielscher Sonifier. Cell debris was removed by centrifugation. The recombinant GST-ArtR protein was isolated from the supernatant using a high-affinity GST resin column (Genscript). Free glutathione in the eluted fractions was removed by dialysis and the purified protein was stored at −70°C. The purity of the protein samples was assessed by SDS-PAGE followed by Coomassie blue staining. Protein concentration was colorimetrically determined by using the Bradford protein assay.

### ATPase Activity Assay

The ATPase activity of GST-ArtR protein was detected by measuring the amount of inorganic phosphate liberated from ATP using NaH_2_PO_4_ as a standard. Experiments were performed in a reaction buffer as described previously (Murat et al., 2006). Purified GST-ArtR protein (0–25 μg) was added into the reaction mixtures. The reaction was started by adding 2 mM ATP and terminated after 1 h by the addition of the Malachite Green reagent. After 5 min, absorbance was measured at 630 nm.

### Subcellular Fractionation

Subcellular fractionation was performed as described previously with minor changes (Casabona et al., 2013). Fifty milliliters of overnight PAO1 culture was centrifuge for 10 min at 2,000 *g*. The cells were washed once with 1 ml of 50 mM Tris-HCl (pH 7.6) and re-suspended in 0.5 ml of 200 mM MgCl_2_, 50 mM Tris-HCl (pH 7.6). After incubation at 30°C for 30 min with gentle shaking, the cells were placed on ice for 5 min, and then incubated for 15 min at room temperature. Centrifuge the cells for 10 min at 8,000 *g* (4°C) and keep supernatant as periplasmic fraction. Wash the cell pellet in 1 ml of 50 mM Tris-HCl (pH 7.6) and centrifugation for 10 min at 8,000 *g* (4°C). Re-suspend the cell pellet in 0.5 ml of 50 mM Tris-HCl (pH 7.6) and sonicate twice for 30 s. Centrifuge at 2,000 *g* for 15 min to remove intact cells and cell debris and take supernatant as cytoplasm and membrane fraction. Transfer the supernatant into a tube compatible with ultracentrifugation and ultracentrifuge for 45 min at 120,000 *g* at 4°C. Harvest the supernatant as the cytoplasmic fraction. Resuspend the pellet corresponding to the total membrane fraction in the desired buffer. The ultrafiltration tube (Millipore) was used for enriching cell fragments, and the fractions then were used in subsequent experiments.

### Confocal Microscopy and Image Acquisition

*Pseudomonas aeruginosa* PAO1 carrying pME6032-*artR-gfp* was cultured with 0.5 mM IPTG at 37°C for 4 h. The cells were examined and imaged by confocal laser scanning microscopy, using a Nikon A1 operating system (Nikon, Japan).

### Western Blot Analysis

Samples from subcellular fractionation were loaded and separated by 12% SDS-PAGE. The proteins were transferred onto a polyvinylidene difluoride (PVDF) membrane and hybridized with a rabbit polyclonal ArtR antibody (Sangon Biotech) generated by using purified His_6_-ArtR protein. The signal was detected by the use of an ECL Plus kit (Amersham Biosciences).

For the test of ExoS and FLAG fusion proteins, overnight cultures of the tested strains were transformed into fresh LB medium (*rsmA*-FLAG) or with 5 mM EGTA and 20 mM MgCl_2_ (ExoS and *exsA*-FLAG) and cultivated to an OD_600_ of 1.0. Hundred microlitres of cultures were centrifuged and the pellets were re-suspended in 10 μl PBS. Bacterial cells were loaded and separated by 12% SDS-PAGE. The proteins were transferred onto a PVDF membrane and hybridized with a rabbit polyclonal ExoS antibody (generated in Dr. Shouguang Jin’s laboratory) or a mouse monoclonal FLAG antibody (Sigma). The signal was detected by an ECL Plus kit (Amersham Biosciences).

### RNA-Seq and Data Analysis

Wild-type strain PAO1 and the Δ*artR* mutant were cultured in LB broth at 37°C and grown to mid-log-phage (OD_600_ = 1.0). Total RNA was isolated with an RNeasy Protect Bacteria minikit (Qiagen) and genomic DNA was eliminated by RNase-free DNase I treatment. After removing rRNA by using the MICROBExpress kit (Ambion), mRNA was used to generate the cDNA library according to the TruSeq RNA sample prep kit protocol (Illumina), which was then sequenced using the HiSeq 2000 system (Illumina). The sequence data were analyzed using a method described previously (Chua et al., 2014).

### Statistical Analysis

Statistical analysis was performed using Student’s *t*-test to determine the statistical significance when applicable: NS*p* > 0.05; ^∗^*p* < 0.05; ^∗∗^*p* < 0.01, and ^∗∗∗^*p* < 0.001.

## Data Availability

The NCBI database accession number is GSE127751.

## Author Contributions

WK and KD designed the study. MD, RY, QL, HZ, WL, YZ, and HL contributed in the acquisition, analysis, and interpretation of the data. WK and KD wrote the manuscript.

## Conflict of Interest Statement

The authors declare that the research was conducted in the absence of any commercial or financial relationships that could be construed as a potential conflict of interest.
