# Supplementary material for: A Unique ATPase, ArtR (PA4595), Represses the Type III Secretion System in Pseudomonas aeruginosa
Source: Front Microbiol. 2019 Mar 21;10:560. doi: 10.3389/fmicb.2019.00560 (PMC6437102; doi:10.3389/fmicb.2019.00560)
Supplement: Supplementary file 1 [file Data_Sheet_1.DOCX]

**Supplementary Material**

**Table S1. Bacterial strains and plasmids used in this study.**

| **Strain or plasmid** | **Phenotype** | **Source or reference** |
| --- | --- | --- |
| ***E. coli*** |  |  |
| DH5α | *F^–^ φ80lacZ ΔM15 Δ(lacZYA-argF)U169 recA1 endA1 hsdR17(rk^–^, mk^+^)phoA supE44 thi-1 gyrA96 relA1 tonA* | Invitrogen |
| SM10-λ *pir* | Mobilizing strain, RP4 integrated in the chromosome; Kn^r^ | (Simon *et al.*, 1983) |
| BL21(DE3)pLysS | *F^–^ ompT gal dcm lon hsdS_B_*(r_B_^-^ m_B_^-^) *λ*(DE3) [*lacI lacUV5*-T7 *gene1 ind1 sam7 nin5*]) pLysS (Cm^r^) | Invitrogen |
| ***P. aeruginosa*** |  |  |
| PAO1 | Wild type |  |
| C1 | *artR* transponson mutant of PAO1; Gm^r^ | This study |
| Δ*artR* | *artR* knockout mutant of PAO1; Gm^r^ | This study |
| (Δ*artR*)C | Δ*artR* complemented strain; Gm^r^, Tc^r^ | This study |
| Δ*exsA* | *exsA* knockout mutant of PAO1; Gm^r^ | (Kong *et al.*, 2013) |
| Δ*exoT* | *exoT* knockout mutant of PAO1; Gm^r^ | (Kong *et al.*, 2013) |
| Δ*exoS* | *exoS* knockout mutant of PAO1; Gm^r^ | (Kong *et al.*, 2013) |
| ΔPA4594 | PA4594 knockout mutant of PAO1; Gm^r^ | This study |
| (Δ*artR*)D187 | Δ*artR* D187E point mutation complemented strain; Gm^r^, Tc^r^ | This study |
| (Δ*artR*)D469 | Δ*artR* D469E point mutation complemented strain; Gm^r^, Tc^r^ | This study |
| (Δ*artR*)D187D469 | Δ*artR* D187E and D469E point mutations complemented strain; Gm^r^, Tc^r^ | This study |
| **Plasmids** |  |  |
| pBT20 | Mini-Tn*M* delivery vector; Gm^r^ | (Kulasekara *et al.*, 2005) |
| pEX18Tc | *oriT*^+^ *sacB*^+^ gene replacement vector with multiple-cloning site from pUC18; Tc^r^ | (Hoang *et al.*, 1998) |
| pZ1918-*lacZ*Gm | Source plasmid of Gm^r^ cassette; Gm^r^ | (Schweizer, 1993) |
| pRK2013 | Broad-host-range helper vector; Tra^+^, Kn^r^ | (Ditta *et al.*, 1980) |
| pMS402 | Expression reporter plasmid carrying the promoterless *luxCDABE* gene; Kn^r^, Tmp^r^ | (Duan *et al.*, 2003) |
| CTX6.1 | Integration plasmid origins of plasmid mini-CTX-*lux*; Tc^r^ | This lab |
| mini-CTX-*lacZ* | Integration plasmid containing *attP* site for integration at chromosomal *attB* site; Tc^r^ | (Becher & Schweizer, 2000) |
| pGEX-4T-1 | GST tag protein expression vector; Ap^r^ | GE Healthcare |
| pAK1900 | *E. coli*-*P. aeruginosa* shuttle cloning vector; Ap^r^, Cb^r^ | (Poole *et al.*, 1993) |
| pEX-*artR* | *artR* knockout plasmid, pEX18Tc with 981 bp upstream region, Gm^r^-*lacZ* fragment from pZ1918-*lacZ*Gm and 1074 bp downstream of *artR* cloned between *Kpn*I and *Hin*dIII; Tc^r^, Gm^r^ | This study |
| pEX-4594 | PA4594 knockout plasmid, pEX18Tc with 439 bp upstream region, Gm^r^-*lacZ* fragment from pZ1918-*lacZ*Gm and 817 bp downstream of PA4594 cloned between *Bam*HI and *Hin*dIII; Tc^r^, Gm^r^ | This lab |
| mini-CTX-*artR* | Complementation plasmid, mini-CTX-*lacZ* with a 2055 bp PCR fragment covering the entire *artR* gene between *Kpn*I and *Hin*dIII; Tc^r^ | This study |
| mini-CTX-*artR*D187 | Complementation plasmid, mini-CTX-*lacZ* with a 2055 bp fragment of *artR* containing D187E point mutation between *Kpn*I and *Hin*dIII; Tc^r^ | This study |
| mini-CTX-*artR*D469 | Complementation plasmid, mini-CTX-*lacZ* with a 2055 bp fragment of *artR* containing D469E point mutation between *Kpn*I and *Hin*dIII; Tc^r^ | This study |
| mini-CTX-*artR*D187D469 | Complementation plasmid, mini-CTX-*lacZ* with a 2055 bp fragment of *artR* containing D187E and D469E point mutations between *Kpn*I and *Hin*dIII; Tc^r^ | This study |
| pGEX-ArtR | ArtR protein expression plasmid, a fragment containing entire ArtR coding region cloned into pGEX-4T-1; Ap^r^ | This study |
| pAK-*artR* | pAK1900 with the entire *artR* gene and the promoter region of *artR*; Ap^r^, Cb^r^ | This study |
| pME6032-*artR-gfp* | pME6032 containing the entire *artR* gene fused with GFP tag at C-terminal; Tc^r^ | This study |
| pKD-*exoS* | pMS402 containing *exoS* promoter region; Kn^r^, Tmp^r^ | (Duan *et al.*, 2003) |
| pKD-*exoY* | pMS402 containing *exoY* promoter region; Kn^r^, Tmp^r^ | (Duan *et al.*, 2003) |
| pKD-*exoT* | pMS402 containing *exoT* promoter region; Kn^r^, Tmp^r^ | (Duan *et al.*, 2003) |
| pKD-*lasR* | pMS402 containing *lasR* promoter region; Kn^r^, Tmp^r^ | (Duan *et al.*, 2003) |
| pKD-*exsC* | pMS402 containing *exsCEBA* promoter region; Kn^r^, Tmp^r^ | (Kong *et al.*, 2013) |
| pKD-*exsD* | pMS402 containing *exsD-pscB-L* promoter region; Kn^r^, Tmp^r^ | (Kong *et al.*, 2013) |
| pKD-*vfr* | pMS402 containing *vfr* promoter region; Kn^r^, Tmp^r^ | This lab |
| pKD-*rsmA* | pMS402 containing *rsmA* promoter region; Kn^r^, Tmp^r^ | This lab |
| CTX-*exoS* | Integration plasmid, CTX6.1 with a fragment of pKD-*exoS* containing *exoS* promoter region and *luxCDABE* gene; Kn^r^, Tmp^r^, Tc^r^ | This lab |
| *exsA*-FLAG-A | pDN19 containing the entire *exsA* gene driven by *tac* promoter fused with FLAG tag at C-terminal; Ap^r^ , Tc^r^ | (Li *et al.*, 2013) |
| *exsA*-FLAG-C | Mini-CTX-*lacZ* containing the entire *exsA* gene driven by *exsCEBA* promoter fused with FLAG tag at C-terminal; Tc^r^ | This lab |
| *rsmA*-FLAG | Mini-CTX-*lacZ* containing the entire *rsmA* gene driven by *rsmA* promoter fused with FLAG tag at C-terminal; Tc^r^ | This lab |

**References**:

Becher, A. & H.P. Schweizer, (2000) Integration-proficient *Pseudomonas aeruginosa* vectors for isolation of single-copy chromosomal *lacZ* and *lux* gene fusions. *Biotechniques* **29**: 948-953.

Ditta, G., S. Stanfield, D. Corbin & D.R. Helinski, (1980) Broad host range DNA cloning system for Gram-negative bacteria: construction of a gene bank of *Rhizobium meliloti*. *Proc Natl Acad Sci U S A* **77**: 7347-7351.

Duan, K., C. Dammel, J. Stein, H. Rabin & M.G. Surette, (2003) Modulation of *Pseudomonas aeruginosa* gene expression by host microflora through interspecies communication. *Mol Microbiol* **50**: 1477-1491.

Hoang, T.T., R.A.R. Karkhoff-Schweizer, A.J. Kutchma & H.P. Schweizer, (1998) A broad-host-range Flp-FRT recombination system for site-specific excision of chromosomally-located DNA sequences: application for isolation of unmarked *Pseudomonas aeruginosa* mutants. *Gene* **212**: 77-86.

Kong, W.N., L. Chen, J.Q. Zhao, T. Shen, M.G. Surette, L.X. Shen & K.M. Duan, (2013) Hybrid sensor kinase PA1611 in *Pseudomonas aeruginosa* regulates transitions between acute and chronic infection through direct interaction with RetS. *Mol Microbiol* **88**: 784-797.

Kulasekara, H.D., I. Ventre, B.R. Kulasekara, A. Lazdunski, A. Filloux & S. Lory, (2005) A novel two-component system controls the expression of *Pseudomonas aeruginosa* fimbrial *cup* genes. *Mol Microbiol* **55**: 368-380.

Li, K.W., C. Xu, Y.X. Jin, Z.Y. Sun, C. Liu, J. Shi, G.K. Chen, R.H. Chen, S.G. Jin & W.H. Wu, (2013) SuhB is a regulator of multiple virulence genes and essential for pathogenesis of *Pseudomonas aeruginosa*. *MBio* **4**: e00419-00413.

Poole, K., S. Neshat, K. Krebes & D.E. Heinrichs, (1993) Cloning and nucleotide sequence analysis of the ferripyoverdine receptor gene *fpvA* of *Pseudomonas aeruginosa*. *J Bacteriol* **175**: 4597-4604.

Schweizer, H.P., (1993) Two plasmids, X1918 and Z1918, for easy recovery of the *xylE* and *lacZ* reporter genes. *Gene* **134**: 89-91.

Simon, R., U. Priefer & A. Pühler, (1983) A broad host range mobilization system for *in vivo* genetic engineering: transposon mutagenesis in Gram negative bacteria. *Nat Biotechnol* **1**: 784-791.

**Table S2. Primers used in this study.**

| **Primer** | **Sequence (5’→3’)^a^** | **Restriction site** |
| --- | --- | --- |
| pEX-*artR*-S | GATGGTACCGTCAGGTCTTGAGCGGCA | *Kpn*I |
| pEX-*artR*-A | GCTAAGCTTGTTCCCATGTTCCCCTGC | *Hin*dIII |
| pEX-4594-S | GCGGGATCCACCTCGCCATGCCGTACTGT | *Bam*HI |
| pEX-4594-A | CGCGAAGCTTCGAAGGCAACTACACCG | *Hin*dIII |
| *artR*D187-A | GTACTCGAGCAGCAGCATGTCGGGAGC | *Xho*I |
| *artR*D187-S | GCACTCGAGGAACCGACCAACCACCTG | *Xho*I |
| *artR*D469-A | GACCTCGAGCAGCAGCACGTTGCC | *Xho*I |
| *artR*D469-S | GAACTCGAGGAACCGTCCAACGACCTC | *Xho*I |
| pGEX-*artR*-S | GATCTCGAGTTGGCTCAGTACGTCTAC | *Xho*I |
| pGEX-*artR*-A | ATTGCGGCCGCTTACGCCAGTTTCTTGTAG | *Not*I |
| *exoS*-qRT-S | CCAGTGAAGCGCAGCAGTTG |  |
| *exoS*-qRT-A | AACAGCTTGCCCAGCCAGTC |  |
| *proC*-qRT-S | CAGGCCGGGCAGTTGCTGTC |  |
| *proC*-qRT-A | GGTCAGGCGCGAGGCTGTCT |  |

^a^Restriction site sequences are underlined.

**Table S3. Comparison of transcript levels between PAO1 and *artR* mutant.**

| **PA No.*** | **Gene name** | **Description** | **Operon context** | **Subcellular localization** | **Fold change (log2)** | |
| --- | --- | --- | --- | --- | --- | --- |
| PA0044 | *exoT* | exoenzyme T |  | Extracellular | 2.78652 |  |
| PA0604 |  | probable binding protein component of ABC transporter | PA0603-PA0604 | Periplasmic | 2.13694 |  |
| PA1706 | *pcrV* | type III secretion protein PcrV | *popN*-*pcr1-H-popB-D* | Extracellular | 3.11984 |  |
| PA1708 | *popB* | translocator protein PopB | *popN*-*pcr1-H-popB-D* | Extracellular | 2.2455 |  |
| PA1709 | *popD* | Translocator outer membrane protein PopD precursor | *popN*-*pcr1-H-popB-D* | Extracellular | 2.42443 |  |
| PA1710 | *exsC* | ExsC, exoenzyme S synthesis protein C precursor | *exsCEB* | Cytoplasmic | 1.9049 |  |
| PA1713 | *exsA* | transcriptional regulator ExsA |  | Cytoplasmic | 2.17281 |  |
| PA1716 | *pscC* | Type III secretion outer membrane protein PscC precursor | *exsD-pscB-L* | Outer Membrane | 2.53011 |  |
| PA1719 | *pscF* | type III export protein PscF | *exsD-pscB-L* | Extracellular | 1.94721 |  |
| PA2191 | *exoY* | adenylate cyclase ExoY |  | Extracellular | 2.52233 |  |
| PA3841 | *exoS* | exoenzyme S |  | Extracellular | 2.42009 |  |
| PA0482 | *glcB* | malate synthase G |  | Cytoplasmic/  Periplasmic | -1.29355 |  |
| PA0494 |  | probable acyl-CoA carboxylase subunit | PA0493-PA0494-PA0495-PA0496 | Cytoplasmic | -2.85878 |  |
| PA0506 |  | probable acyl-CoA dehydrogenase |  | Cytoplasmic/  Periplasmic | -1.14405 |  |
| PA0519 | *nirS* | nitrite reductase precursor | *nirS-nirM-nirC-nirF-*PA0515*-nirL-*PA0513-PA0512-*nirJ-*PA0510*-nirN* | Periplasmic | -1.61325 |  |
| PA0524 | *norB* | nitric-oxide reductase subunit B | *norC-norB-*PA0525 | Cytoplasmic Membrane | -4.52793 |  |
| PA0588 | *yeaG* | conserved hypothetical protein | PA0588-PA0587-PA0586 | Cytoplasmic | -1.68759 |  |
| PA0795 | *prpC* | citrate synthase 2 | PA0797*-prpB-prpC* | Cytoplasmic | -2.15315 |  |
| PA0872 | *phhA* | phenylalanine-4-hydroxylase | *phhA-phhB-phhC* | Cytoplasmic | -1.41825 |  |
| PA1041 |  | probable outer membrane protein precursor |  | Outer Membrane | -1.82866 |  |
| PA1074 | *braC* | branched-chain amino acid transport protein BraC |  | Periplasmic | -1.40395 |  |
| PA1155 | *nrdB* | NrdB, tyrosyl radical-harboring component of class Ia ribonucleotide reductase |  | Cytoplasmic/  Periplasmic | -1.13579 |  |
| PA1178 | *oprH* | PhoP/Q and low Mg2+ inducible outer membrane protein H1 precursor | *oprH-phoP-phoQ* | Periplasmic/  Outer Membrane | -1.18703 |  |
| PA1249 | *aprA* | alkaline metalloproteinase precursor |  | Extracellular | -1.63776 |  |
| PA1337 | *ansB* | glutaminase-asparaginase | *ansB*-PA1336-PA1335 | Periplasmic | -2.32858 |  |
| PA1342 |  | probable binding protein component of ABC transporter |  | Periplasmic | -1.62904 |  |
| PA1903 | *phzE2* | phenazine biosynthesis protein PhzE | *phzB2-G2* | Cytoplasmic | -2.73104 |  |
| PA1984 | *exaC* | NAD+ dependent aldehyde dehydrogenase ExaC | *exaB-exaC* | Cytoplasmic/  Periplasmic | -1.79589 |  |
| PA2008 | *fahA* | fumarylacetoacetase | *hmgA-fahA-maiA* | Cytoplasmic | -1.95049 |  |
| PA2009 | *hmgA* | homogentisate 1,2-dioxygenase | *hmgA-fahA-maiA* | Periplasmic | -1.52661 |  |
| PA2247 | *bkdA1* | 2-oxoisovalerate dehydrogenase (alpha subunit) | *bkdA1-bkdA2-bkdB-lpdV* | Cytoplasmic | -1.81654 |  |
| PA2248 | *bkdA2* | 2-oxoisovalerate dehydrogenase (beta subunit) | *bkdA1-bkdA2-bkdB-lpdV* | Cytoplasmic | -2.09387 |  |
| PA2291 | *oprB2* | probable glucose-sensitive porin |  | Outer Membrane | -1.711 |  |
| PA2552 | *acdB* | probable acyl-CoA dehydrogenase | PA2555-PA2552 | Cytoplasmic | -1.95097 |  |
| PA2623 | *icd* | isocitrate dehydrogenase |  | Cytoplasmic/  Periplasmic | -1.34455 |  |
| PA2788 |  | probable chemotaxis transducer |  | Cytoplasmic Membrane/  Outer Membrane | -1.89476 |  |
| PA2939 |  | probable aminopeptidase |  | Extracellular | -2.79646 |  |
| PA3013 | *foaB* | fatty-acid oxidation complex beta-subunit | *faoA-foaB* | Cytoplasmic | -1.23921 |  |
| PA3014 | *faoA* | fatty-acid oxidation complex alpha-subunit | *faoA-foaB* | Cytoplasmic/  Periplasmic | -1.19794 |  |
| PA3068 | *gdhB* | NAD-dependent glutamate dehydrogenase |  | Cytoplasmic | -1.05217 |  |
| PA3186 | *oprB* | Glucose/carbohydrate outer membrane porin OprB precursor | PA3189-PA3188-PA3187-*oprB* | Outer Membrane | -2.62028 |  |
| PA3187 | *gltK* | probable ATP-binding component of ABC transporter | PA3189-PA3188-PA3187-*oprB* | Cytoplasmic Membrane | -2.03377 |  |
| PA3188 | *gltG* | probable permease of ABC sugar transporter | PA3189-PA3188-PA3187-*oprB* | Cytoplasmic Membrane | -2.46745 |  |
| PA3190 | *gltB* | probable binding protein component of ABC sugar transporter |  | Periplasmic | -2.26304 |  |
| PA3250 |  | hypothetical protein | PA3250-PA3255 | Periplasmic | -4.11393 |  |
| PA3280 | *oprO* | Pyrophosphate-specific outer membrane porin OprO precursor |  | Outer Membrane | -2.41373 |  |
| PA3326 | *clpP2* | ClpP2 |  | Cytoplasmic | -1.1838 |  |
| PA3391 | *nosR* | regulatory protein NosR | *nosR-nosZ-nosD-nosF-nosY-nosL* | Cytoplasmic Membrane | -4.94331 |  |
| PA3392 | *nosZ* | nitrous-oxide reductase precursor | *nosR-nosZ-nosD-nosF-nosY-nosL* | Periplasmic | -4.92584 |  |
| PA3554 | *arnA* | ArnA | *arnB-arnC-arnA-arnD-arnT-arnE-arnF*-PA3559 | Cytoplasmic | -1.79133 |  |
| PA3570 | *mmsA* | methylmalonate-semialdehyde dehydrogenase | *mmsA-mmsB* | Cytoplasmic | -2.62239 |  |
| PA3692 | *lptF* | Lipotoxon F, LptF | PA3691-*lptF* | Outer Membrane | -1.26142 |  |
| PA3724 | *lasB* | elastase LasB |  | Extracellular | -1.42714 |  |
| PA3790 | *oprC* | Putative copper transport outer membrane porin OprC precursor | *oprC*-PA3789 | Outer Membrane | -2.09777 |  |
| PA3795 |  | probable oxidoreductase |  | Cytoplasmic | -1.49373 |  |
| PA3922 |  | conserved hypothetical protein | PA3923-PA3922 | Periplasmic | -1.99677 |  |
| PA4022 | *hdhA/* *exaC2* | hydrazone dehydrogenase, HdhA |  | Cytoplasmic | -2.01596 |  |
| PA4133 | *ccoN* | cytochrome c oxidase subunit (cbb3-type) | PA4133-PA4134 | Cytoplasmic Membrane | -2.33704 |  |
| PA4211 | *phzB1* | probable phenazine biosynthesis protein | *phzB1-phzG1* | Cytoplasmic | -1.96653 |  |
| PA4214 | *phzE1* | phenazine biosynthesis protein PhzE | *phzB1-phzG1* | Cytoplasmic | -2.68138 |  |
| PA4217 | *phzS* | flavin-containing monooxygenase |  | Cytoplasmic | -2.839 |  |
| PA4236 | *katA* | catalase |  | Periplasmic | -1.1312 |  |
|  |  |  |  |  |  |  |
| PA4595 | *yjjK* | probable ATP-binding component of ABC transporter |  | Cytoplasmic | -2.77774 |  |
| PA4607 |  | hypothetical protein |  |  | -1.75569 |  |
|  |  |  |  |  |  |  |
| PA4773 |  | hypothetical protein |  |  | -2.35938 |  |
| PA4774 |  | hypothetical protein | PA4774-PA4775*-pmrA-pmrB* | Cytoplasmic | -1.66517 |  |
| PA5178 |  | conserved hypothetical protein |  | Outer Membrane | -1.17361 |  |
| PA5360 | *phoB* | two-component response regulator PhoB | *phoB-phoR-*PA5362 | Cytoplasmic | -1.95439 |  |
| PA5367 | *pstA* | membrane protein component of ABC phosphate transporter | *pstC-pstA-pstB* | Cytoplasmic Membrane | -2.24422 |  |
| PA5368 | *pstC* | membrane protein component of ABC phosphate transporter | *pstC-pstA-pstB* | Cytoplasmic Membrane | -2.73784 |  |
| PA5369 | *pstS* | phosphate ABC transporter, periplasmic phosphate-binding protein, PstS |  | Cytoplasmic Membrane/  Periplasmic | -2.94672 |  |

*Only the genes that showed more than 2-fold changes of expression are listed**.**





**Figure S1. Expression of ExoS in PAO1 and Δ*artR*.** Bacteria were grown to an OD_600_ of 1.0 in LB with or without 5 mM EGTA and 20 mM MgCl_2_. Intracellular ExoS from equivalent number bacterial cells was electrophoresed on SDS-PAGE and probed with an anti-ExoS antibody.

**

**

**Figure S2. Phylogenetic relationship of the REG subfamily of ATPases in *P. aeruginosa* PAO1 and** ***E. coli* K-12.** The phylogenetic tree was constructed using MEGA 7.0 by the neighbor-joining method.

**Figure S3. Sequence alignment of the REG subfamily ATPases ArtR and EttA.** The alignment was generated using DNAMAN. ArtR has 74.77% similarity to EttA.


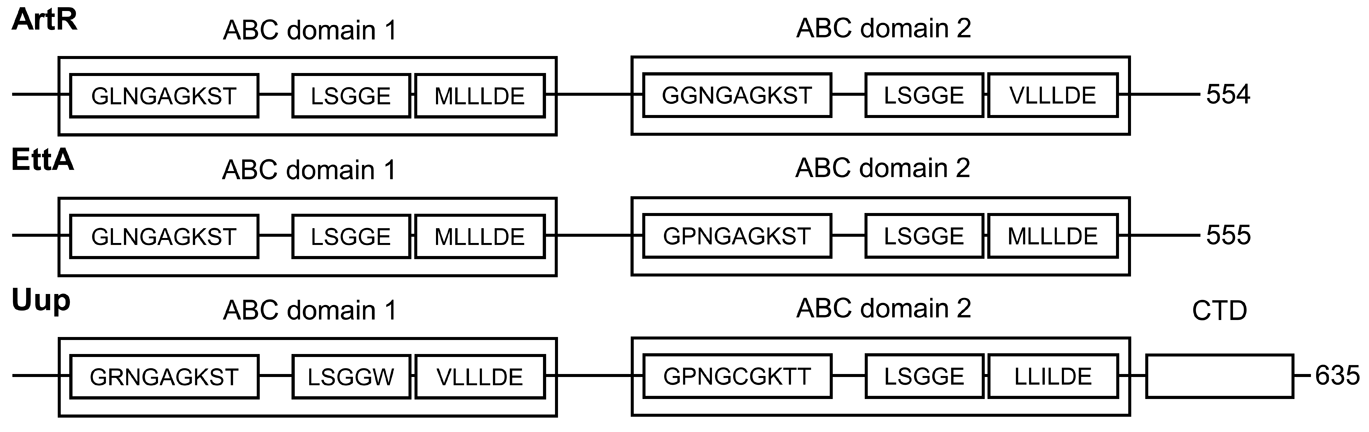


**Figure S4. Domain organization of ArtR, EttA and Uup.** The proteins are represented in a linear fashion (domain sizes not to scale). Conserved Walker motifs A and B and the ABC signature are indicated within rectangles.





**Figure S5. IPTG-dependent overexpression of GST**-**ArtR protein.** BL21(DE3)pLysS cells carrying pGEX-ArtR were cultured in 5 ml of LB medium at 37℃, and solubility of the GST-ArtR protein inducing by IPTG was tested. lane 1, un-induced cells; lane 2, IPTG-induced cells; lane 3, soluble protein of IPTG-induced cells; lane 4, non-soluble protein of IPTG-induced cells; lane 5-6, purified of GST-ArtR protein.
